# Stem Cell-Based Therapies for Liver Diseases: State of the Art and New Perspectives

**DOI:** 10.4061/2010/259461

**Published:** 2010-09-01

**Authors:** Anna Chiara Piscaglia, Mariachiara Campanale, Antonio Gasbarrini, Giovanni Gasbarrini

**Affiliations:** “Gastrointestinal and Liver Stem Cell Research Group” (GILSteR), Department of Internal Medicine, Gemelli Hospital, Catholic University of Rome, Largo A. Gemelli 8-00168 Roma, Italy

## Abstract

Millions of patients worldwide suffer from end-stage liver pathologies, whose only curative therapy is liver transplantation (OLT). Given the donor organ shortage, alternatives to OLT have been evaluated, including cell therapies. Hepatocyte transplantation has been attempted to cure metabolic liver disorders and end-stage liver diseases. The evaluation of its efficacy is complicated by the shortage of human hepatocytes and their difficult expansion and cryopreservation. Recent advances in cell biology have led to the concept of “*regenerative medicine*”, based on the therapeutic potential of stem cells (SCs). Different types of SCs are theoretically eligible for liver cell replacement. These include embryonic and fetal SCs, induced pluripotent cells, annex SCs, endogenous liver SCs, and extrahepatic adult SCs. Aim of this paper is to critically analyze the possible sources of SCs suitable for liver repopulation and the results of the clinical trials that have been published until now.

## 1. Cell Therapies for Liver Diseases

Liver pathologies affect hundreds of millions of patients worldwide. The most common causes of hepatopathy are chronic hepatitis C and B, alcoholism, nonalcoholic fatty liver disease, autoimmune, and drug-induced hepatic disorders. Many of these conditions can be prevented or treated, but if not, they can lead to progressive liver injury, liver fibrosis and ultimately cirrhosis, portal hypertension, liver failure, and, in some instances, cancer. There are currently more than 5 million people in the United States suffering from end-stage liver pathologies, whose only curative therapy is liver transplantation (OLT). More than 5,000 liver transplants are performed in the United States each year (including more than 500 in children). About 20,000 people are waiting for OLT, but only 7,000 transplants are performed annually and as many as 1,500 patients die yearly while on the waiting list [1]. 

Given the donor organ shortage, various alternatives to OLT have been evaluated, such as split-liver and related living-donor liver transplantation. These procedures are still limited by the donor scarcity, the high costs, and the lifelong immunosuppressive treatments that they all require [2, 3]. Thus, the development of cell therapies for the treatment of end-stage hepatic diseases is currently under investigation all over the world. A cell therapy can be defined as *“the use of living cells to restore, maintain, or enhance tissue and organ function”* [4]. Cell therapies in hepatology have numerous potential advantages when compared to OLT, since transplantable cells can be (1) *in vitro *expanded and cryopreserved, abolishing the limit of organ shortage; (2) genetically manipulated, to correct inborn errors of metabolism; (3) cryopreserved for future use; (4) infused without major surgery; (5) obtained from the same patient, avoiding risk of rejection and need for lifelong immunosuppression [5]. 

One possible cell therapy source to restore the liver functional mass is represented by adult hepatocytes, that represent a particularly appealing tool, because they are mature and fully functional hepatic cells. Since the first successful hepatocyte transplantation in a rodent model of Crigler-Najjar syndrome, many preclinical studies and clinical applications of this technique have been made to cure metabolic liver disorders and end-stage liver diseases [6]. In most instances, hepatocyte transplantation has been able to grant a clinical improvement for up to 12 months [7]. In patients with liver failure, hepatocyte-based therapies have also included the use of human or porcine hepatocytes in bioartificial liver devices [8]. Despite some encouraging results, the interpretation of these studies is hampered by the limited number and heterogeneity of patients, the lack of controls, the variety in terms of experimental design, outcome parameters, and follow-up duration. The evaluation of the efficacy of hepatocyte transplantation and bioartificial liver support systems is further complicated by the shortage of human hepatocytes. Indeed, it is ethically difficult to assign cadaveric livers to these experimental protocols, while many patients still die on the OLT waiting list. Moreover, primary cultured hepatocytes are hard to expand *in vitro* and cryopreserved cells are easily damaged during the freezing-thawing procedure. As a consequence, alternative solutions are being examined in the hepatic cell therapy field. Among these, of particular interest is the so-called *“regenerative medicine”*, based on the therapeutic potential of stem cells (SCs) [5, 9]. 

SCs are undifferentiated cells, able to give rise to diverse mature progenies and to self-renew, through the alternation of symmetrical and asymmetrical divisions. SCs exist in all multicellular organisms and play a central role in tissue genesis, regeneration, and homeostasis, by providing new elements to increase tissue mass during pre- and postnatal growth, and by replacing cell loss due to senescence or damage [10, 11]. SCs possess a hierarchy of potentialities: from the totipotency of the zygote and its immediate progeny, to the pluripotency of embryonic stem cells (ESCs), to the multi/unipotency of tissue-specific adult SCs (ASCs). The latter persist in terminally differentiated tissues, allowing for their renewal and regeneration [12–15]. SCs colocalize with supporting cells in a physiologically limited and specialized microenvironment, or “*niche*”, that varies in nature and location depending upon the tissue type [16]. The reciprocal interactions between SCs and their microenvironment, through cell-cell and cell-matrix connections as well as the secretion of soluble factors, influence SC behavior [17, 18]. 

SCs are already leaving the bench and reaching the bedside, despite an incomplete knowledge of the genetic control program driving their fate and plasticity [5]. In hepatology, the first attempts to translate SC basic research into new clinical strategies have been made. Aim of this review is to summarize the state of the art on SC-based therapies in hepatology. In particular, we will discuss the possible sources of SCs suitable for liver repopulation and will highlight both the benefits and the potential risks of these new tools for the treatment of liver pathologies.

## 2. Embryonic, Fetal, and Annex Stem Cells

### 2.1. Embryonic and Fetal Liver Stem Cells

ESCs and their derivatives might constitute an easily available source to obtain a large number of transplantable cells for regenerative treatments. ESCs are pluripotent cells derived from the inner cell mass of the blastocyst and can generate any differentiated phenotype of the three primary germ layers (endoderm, mesoderm, and ectoderm), as well as germ cells [15]. ESCs can be indefinitely maintained in an undifferentiated state, though they seem to develop karyotypic abnormalities over long periods in culture [5]. It has been demonstrated that ESCs can differentiate *in vitro* towards the hepatic lineage by simple removal of factors that prevent their differentiation, and/or through the exposure to appropriate growth factors, as reviewed elsewhere [7, 19]. Moreover, in several animal models of hepatic disease, ESC-derived hepatocyte-like cells were able to colonize the injured liver and function as mature hepatocytes [7, 19]. Fetal liver SCs, also named “*hepatoblasts”*, appear when the hepatic endoderm has been specified and the liver bud is growing. Hepatoblasts are bipotent, being able to give rise to both hepatocytes and bile duct cells, and coexpress biliary and hepatocytic markers, such as albumin, alpha-fetoprotein (AFP), and CK19. Studies in diverse model organisms have revealed evolutionarily conserved inductive signals and transcription factor networks that elicit the differentiation of liver SCs, as reviewed elsewhere [19]. Murine hepatoblast cell lines have been established by various research groups and their capacity to repopulate the liver upon transplantation in animal models has been extensively proved, as discussed elsewhere [20]. In contrast to adult liver, ESCs and fetal liver SCs are thought to be highly proliferative, less immunogenic and more resistant to cryopreservation. However, ethical issues and the possibility of immune rejection and teratoma/teratocarcinoma formation in the recipients explain why their use is currently reserved to preclinical studies [21].

### 2.2. Induced Pluripotent Stem Cells

The recently described induced pluripotent stem cells (iPSs) might circumvent the ethical concerns and the risk of rejection related to embryonic and fetal liver SCs. Indeed, iPSs are embryonic-like SCs derived from somatic cells by forced expression of reprogramming factors (Oct3/4 and Sox2 along with either Klf4 or Nanog and Lin28). Theoretically, iPSs could be obtained from the same patient and used for tissue replacement or gene therapy. It is not yet clear how precisely the known developmental signals must be orchestrated to properly program hepatic cells at will, but detailed studies of the activated signaling pathways and their cross-regulatory interactions during embryogenesis will be informative. The first step of hepatic development from iPSs is the induction of definitive endoderm by using activin A. Further treatment with BMP-4 and bFGF can then direct cells towards the hepatic lineage [19]. Nowadays, iPS-based cell therapies have been applied in several animal models of pathologies, with encouraging results, and human iPS cells have been demonstrated to possess a hepatocyte-lineage differentiation potential comparable to that of ESCs [22]. Even if some limitations still remain (i.e., the potential for teratoma formation), iPS-derived hepatocytes are a very promising population for cell therapies in hepatology. 

### 2.3. Annex Stem Cells

Another promising source for SC-based treatments in hepatology may be represented by cells established from placental/cordonal tissues, which do not seem to form teratomas or teratocarcinomas in humans, and have higher proliferation and differentiation potential than ASCs. Several studies indicated that umbilical cord and umbilical cord blood, placenta and amniotic fluid are an easily accessible source of pluripotent SCs, which may be readily available for transplantation, or for further expansion and manipulation prior to cell therapies [18, 23]. These cells can be extensively expanded without loss of potency and have a broad differentiation potential, since they can generate progenies of all three germ layers. These pluripotent annex SCs can be forced to differentiate into hepatocyte-like cells *in vitro* and are capable of liver repopulation *in vivo*, upon transplantation in animal models [7, 24]. We demonstrated that human umbilical cord blood SCs were able to colonize the liver and differentiate into hepatocytes after acute toxic liver damage in NOD/SCID mice and in immunocompetent rats [25, 26]. Moreover, microarray analysis led us to the identification of genes whose modulation strongly correlated with a more efficient process of liver repair after SC injection, proving the ability of these cells to positively influence the hepatic microenvironment and enhance the endogenous hepatic regeneration process [27]. The plasticity and accessibility of cord blood SCs have given the rationale for the creation of cord blood unit banks, where these cells can be collected and stored for future use.

## 3. Endogenous Adult Liver Stem/Progenitor Cells

Regenerative processes in postnatal liver parallel those occurring in development and involve populations of SCs and progenitor cells that can be identified by anatomic, antigenic, and biochemical profiles [28]. In particular, the liver has an extensive regenerative potential in response to parenchymal loss, mainly granted by mature hepatocytes, which can re-enter the cell cycle to restore the liver mass. This is a very efficient system and, after 2/3 partial hepatectomy (PH) in rats, proliferation of hepatocytes and cholangiocytes, followed by stellate and endothelial cells, can regrow the remnant to the original mass in less than 2 weeks [29]. However, whenever the replication ability of hepatocytes is experimentally inhibited or impaired by advanced chronic injury, liver regeneration can still be accomplished by the activation, expansion, and differentiation of the so-called “*hepatic progenitor/stem cells”. *These cells are thought to be responsible for the human ductular reaction, which corresponds to the oval cell activation seen in specific rodent models of liver injury [30]. 

The term “*oval cells”* (OCs) was introduced to describe small proliferating cells with oval nuclei observed in rat livers following certain carcinogenic regimens [17, 31, 32]. OCs are an extremely heterogeneous population, which includes various fractions with different stemness potential, depending upon the experimental protocol and the animal model under investigation [33]. OCs are bipotent (able to give rise to both cholangiocytes and hepatocytes) and coexpress biliary and hepatocytic markers, such as CK19, alpha-fetoprotein, and albumin [34]. Over the past thirty years, several studies have described the isolation, culture differentiation and *in vivo* transplantation of liver OCs. A variety of surface antigens—including OV6, CD44, EpCAM and hematopoietic markers (i.e., CD34, CD133, c-kit)—have been used to identify and isolate OCs, as reviewed elsewhere [5, 28, 35]. Recently, Grompe's group has generated a new collection of monoclonal antibodies by immunization of Fischer rats with enzymatically dispersed nonparenchymal cells from the livers of adult mice treated with 3,5-diethoxycarbonyl-1,4-dihydrocollidine, to produce cell surface reactive reagents more specific for the oval cell response. Differential activity was observed on normal liver cells and at different stages of oval cell activation, indicating potential utility for progenitor cell identification [36]. Recently, Kamiya et al. were able to enrich and culture CD133^+^/CD13^+^/CD49f^+^ bipotent progenitor cells from noninjured postnatal livers [37], while Sackett et al. have proposed the winged helix transcription factor Foxl1 as a potential marker of OCs in mice [38]. 

In humans, the counterpart of OCs is represented by the *intermediate hepatobiliary cells,* or *ductular hepatocytes,* or *hepatic progenitor cells* (HPCs), which can be seen in several hepatopaties, such as chronic cholestasis, submassive necrosis, alcoholic liver disease, focal nodular hyperplasia, and liver allograft failure [39]. In such conditions, intermediates between hepatic SCs and hepatoblasts and between hepatoblasts and adult parenchyma are observed and amplification of one or both pluripotent cell subpopulations can occur [28]. A frequent trigger of ductular reaction is the presence of chronic damage, resulting in hepatocyte senescence. Like OCs, HPCs are bipotent, coexpress biliary and hepatocytic markers, and also express hematopoietic progenitor cell antigens [40, 41]. In the last years, numerous studies have been published on the isolation, characterization and differentiation of putative liver progenitor cells from human livers, but the identification of a specific marker of HPCs awaits further investigation [5, 33, 37, 42–44]. 

Overall, whether OCs/HPCs fulfil the criteria to be considered true LSCs is still controversial. Some authors believe that OCs and HPCs may represent transit-amplifying cells derived from a more primitive LSC [32]. Given the difficulty of identifying unique LSC markers, it has been suggested to consider *stem*-*cellness *as a function instead of an entity: marking resident SCs based upon their quiescence might differentiate true SCs from their rapidly dividing derivatives [5]. By using a label-retention assay following acetaminophen-induced liver injury in mice, Kuwahara et al. found 4 possible LSC populations [45]: (a) replicating hepatocytes at the parenchymal/stromal interface; (b) ductular cells of the canal of Hering (CoH); (c) cholangiocytes of the intralobular bile ducts; and (d) periductular *null cells* (devoid of hepatocytic and biliary markers). The asymmetrically dividing cells that populate these sites might represent some form of lineage hierarchy within the LSC population: periductular null cells might give rise to the cytokeratin-positive cells of the CoH, which in turn could give rise to the intraductular cells and periductular hepatocyte-like cells [46]. Recently, De Alwis et al. have demonstrated that regenerating hepatocytes arise from a LSC population in the CoH and move outward into the liver parenchyma, as in the *streaming liver hypothesis*. Interestingly, this observation seems to indicate that the LSC population is active also in healthy livers and contributes to the hepatic turnover [47]. In conclusion, LSC participation to hepatic repair is probably more complicated than in organs with normally rapid cell turnover and multiple liver cell populations might function as LSCs, depending on severity, location, and chronicity of injury. 

The existence of multiple populations of liver cells with stemness potential implies the existence of multiple LSC niches, that can be activated depending upon the mechanism and location of injury [45, 46]. Presumably, the damaged liver releases molecules that stimulate the activation of OCs/HPCs and mediate their subsequent proliferation, migration and differentiation into mature hepatic phenotypes. Up to date, an extensive number of growth factors and cytokines which regulate the various phases of the OCs/HPCs response have been described, including SCF, HGF, EGF, FGF, Hedgehog and Wnt signalling pathways, SDF-1/CXCR4 axis, tumor necrosis factor, interleukin-6, interferons, transforming growth factors, and transforming growth factor like weak inhibitor of apoptosis (TWEAK) [48, 49]. Noteworthy, the responses of OCs/HPCs to HGF *via* cMet and the potential autocrine loops with TGF-alpha, EGF and FGF are similar to the patterns expressed by hepatocytes during liver regeneration, despite the fact that hepatocytes and LSCs do not tend to proliferate contemporaneously. This might be due to the modulatory effects of inflammatory cells within the niche, producing a range of cytokines and chemokines (such as TWEAK, TGF-beta and INF-gamma) that could influence the LSC response [50]. Another molecule of growing interest in the field of liver regeneration is the granulocyte-colony stimulating factor (G-CSF), a cytokine involved in mediating hematopoietic stem cells (HSCs) mobilization from the bone marrow (BM) into the peripheral blood [51]. In the last decade, several studies have indicated that G-CSF may be effective in mobilizing BM cells that contribute to liver repair [5, 51]. In 2007, we elucidated the double mechanism of action of G-CSF during OC-mediated liver regeneration in rats: G-CSF is able to contribute to liver repair by increasing the BM-derived liver repopulation *(vide infra),* and also by activating the endogenous OCs, that express G-CSF receptor (G-CSFR) [52]. The upregulation of G-CSFR subsequent to G-CSF administration has been recently observed in a small-for-size liver model, confirming a direct receptor-mediated effect of G-CSF on the hepatic parenchyma [53]. These data expand the knowledge regarding the spectrum of actions of G-CSF on ASCs [51, 52]. In fact, it has been demonstrated that G-CSF and its receptor are widely present in neurons and adult neural stem cells. This expression is induced by ischemia, and both counteracts neuronal degeneration and contributes to long-term plasticity after damage. Similarly, G-CSF administration following myocardial infarction in mice induces G-CSFR expression in cardiomyocytes and results in the prevention of cardiac remodeling.

In summary, despite the efforts toward the characterization of human LSCs and of their niches, in view of a possible use for cell therapies, the microenvironmental factors driving LSC fate and LSCs themselves need to be univocally defined prior to attempting any clinical application. The identification of endogenous LSCs and of the signals that govern their proliferation and differentiation to mature hepatocytes might lead to the development of clinically feasible methods to induce liver repopulation from these endogenous cells and/or to allow maturation of stem/progenitor cells to hepatocytes *in vitro* and *in vivo*. 

## 4. Extrahepatic Adult Stem Cells with Hepatogenic Potential

Liver regeneration is mainly an endogenous process, driven by hepatocytes and resident hepatic stem/progenitor cells. However, it has been observed that certain populations of extrahepatic ASCs can migrate into the liver and contribute to its repopulation and turnover [5]. A particularly high degree of plasticity has been shown by bone marrow stem cells (BMSCs), which can give rise to a wide range of phenotypes, including hepatocyte-like cells. Since the pioneering study by Petersen et al. in 1999 [54], numerous reports and reviews have been published on BM contribution to liver regeneration, often with contradictory conclusions [23, 55, 56]. It is generally agreed that BM represents a possible source of LSCs, even if the frequency of colonization, in the absence of a strong selective pressure, is very low, unlikely sufficient *per se* to achieve a significant contribution to hepatic repopulation. However, the few BM-derived cells which do engraft may play an important role in modulating the endogenous repair mechanisms within the hepatic stem cell niche [5, 27, 57]. As clearly demonstrated by Petersen's and our group in 2007, BMSCs may or may not play a critical role in liver regeneration, depending upon the experimental setting [58]. Based on the already cited model of lineage hierarchy within the LSC population [46], we can postulate that the periductular null cells might, at least in part, originate from extrahepatic SCs of BM origin and then give rise to the ductular cells of the CoH. These cells, in turn, can differentiate into intraductular cells and periductular hepatocyte-like cells. 

Regarding the mechanisms underlying BMSC plasticity, upon engraftment BMSCs might either transdifferentiate into parenchymal cells or fuse with resident cells in the host tissue. Fusion phenomena between BMSCs and other cell types (i.e., Purkinjie cells, cardiomyocytes and hepatocytes) have been shown both *in vitro* and *in vivo* [5]. Cell fusion is a physiological phenomenon in certain districts, such as liver and muscle, and it may or may not play a prominent role in SC plasticity, depending on the model of injury and the host phenotype [59]. It has been also proved that fusion and transdifferentiation can coexist and produce therapeutically beneficial results [60, 61]. 

In order to initiate a BM response, the injured liver must signal to the responding cell types. A pivotal role in BMSC recruitment is played by SDF-1. BM-derived liver-committed SCs expressing SDF-1 receptor (CXCR4) are present in the peripheral blood and may colonize the damaged liver by following a SDF-1 gradient [62, 63]. Other molecules secreted by the injured hepatic milieu that can contribute to BMSC recruitment and homing into the liver are the hepatocyte growth factor (HGF), some fibrosis mediators, such as matrix metalloproteinase-9 (MMP9), and the G-CSF [64–66]. The mechanisms underlying the adhesion and retention of BMSCs to human liver compartments have been only in part elucidated [67]. 

Adult BM comprises two main populations of ASCs able to convert into hepatic cells, either by fusion or transdifferentiation: hematopoietic SCs (HSCs) and mesenchymal stem cells (MSCs). 

HSCs are responsible for the renewal of blood cells and can be also isolated from umbilical cord blood and peripheral blood. It is generally accepted that the most primitive and long-term human HSCs are characterized by the expression of CD133, Thy1 (CD90) and VEGFR2 and by a variable expression of CD34 and CD38 [68]. BM-resident HSCs can be mobilized into the peripheral blood under specific stimuli such as tissue injury or administration of G-CSF [5]. Mobilized HSCs can colonize extramedullar sites and participate to their regeneration, by promoting the immune response and/or by transdifferentiating into ASCs within peripheral tissues [5, 18, 62].

MSCs, also called *stromal stem cells, mesenchymal progenitors, mesenchymal stromal cells, colony-forming unit-fibroblastic cells,* are highly proliferating, adherent cells, that reside in a perivascular BM niche and also in the wall of blood vessels within most organs [69]. Numerous studies have demonstrated that MSCs are able to differentiate into a variety of mesodermal cell lineages (osteoblasts, chondroblasts, adipocytes, myocytes, and cardiomyocytes), as well as nonmesodermal cells (such as hepatocytes and neurons), depending upon the microenvironment in which they reside [70]. MSCs might become a more suitable source for SC-based therapies than HSCs, because of their immunological properties: MSCs are less immunogenic and can induce tolerance upon transplantation [71]. Moreover, MSCs showed the highest potential for liver regeneration compared with other BM cell subpopulations in an animal model of hepatic injury [72]. 

A more recently identified SC population within the BM, the so-called *multipotent adult progenitor cells* (MAPCs), seems to be endowed with an impressive plasticity and has shown liver differentiation potential both in culture and in animal models [57]. These cells could potentially copurify with HSCs or MSCs and contaminate these cell populations investigated in liver repopulation studies. According to this hypothesis, rather than being a source of liver-committed SCs, BM could act as a hide out for recirculating pluripotent SCs that might be deposited early during development in BM and could be a source for tissue/organ regeneration [62, 73]. Therefore, the present distinction between HSCs and MSCs may become obsolete, given the heterogeneity and possible overlaps of these various BMSC populations, which could share a common *stem cellness* core [5]. 

As a closing remark, it is worth a note that adipose tissue has been reported as a rich source of easily accessible MSCs (*adipose tissue stromal cells*, ATSCs) capable of hepatic differentiation *in vitro *and* in vivo *[74, 75]. ATSCs are similar to BM-MSCs in terms of surface antigen marker profile and differentiation potential, and ATSCs have been reported to exert an even higher proliferative capacity *in vitro *[76, 77]. We have recently achieved the hepatogenic conversion of ATSCs, using a two-step protocol with sequential addition of growth factors. In order to understand the molecular events involved in ATSC hepatic transdifferentiation, the full genome expression profiles of ATSC-derived hepatocyte-like cells *versus* undifferentiated ATSCs were compared. We identified several targets that depict the numerous biological functions exerted by the liver, including protein metabolism, innate immune response regulation, and biodegradation of toxic compounds. Moreover, we showed that ATSC differentiation into hepatocyte-like cells might be caused by a mesenchymal-to-epithelial transition [78].

Overall, despite an incomplete knowledge of their biological properties, the plasticity and accessibility of HSCs and MSCs from BM and adipose tissue render these ASCs an attractive tool for the regenerative medicine.

## 5. Stem Cell-Based Therapies in Hepatology

As previously discussed, different types of SCs with hepatic differentiation potential are theoretically eligible for liver cell replacement. These include ESCs, iPSs, hepatoblasts, annex SCs, and adult SCs, such HPCs, HSCs, and MSCs. Despite encouraging results *in vitro*, the use of hepatocyte-like cells derived from these stem/progenitor cell populations is still confined to preclinical studies, given the scarce tissue-specific functionality and, up to now, the lack of evidence of strong liver repopulation levels in animal models. Nowadays, the most promising source for SC-based therapies is represented by the intraportal or intrahepatic infusion of freshly isolated or *in vitro* expanded HSCs [79]. Another appealing option is represented by the administration of mobilizing/proliferating agents, such as G-CSF, that is able to both enhance the HSC mobilization into the peripheral blood and facilitate the endogenous LSC activation [5]. G-CSF administration could also exert beneficial immunomodulatory effects in presence of liver failure, since it can reverse the neutrophil defects and the status of *immune paralysis* associated with severe hepatic insufficiency [49, 50, 80].

### 5.1. Bone Marrow Stem Cell Transplantation

BMSCs seem to be physiologically involved in liver repair in humans. A spontaneous mobilization of CD34^+^ cells has been reported following liver resection in patients with primary liver cancer or metastasis [81]. Similarly, a significant increase in the percentage of CD133^+^ cells has been found by Gehling et al. in blood samples of healthy living liver donors and further *in vitro* investigations have demonstrated that the mobilized cells were indeed liver committed [82]. Recently, the same authors observed that liver cirrhosis is associated with an intermittent mobilization of various populations of liver-committed cells of putative BM origin into the circulation [83]. 

The possible therapeutic interest of BMSCs in hepatology was firstly investigated in 2005: intraportal autologous transplantation of CD133^+^ BMSCs in patients with liver cancer undergoing portal embolization before extensive liver resection (LR) achieved some clinical improvement [84]. Similar results were obtained two years later by Fürst et al. who concluded that in patients with malignant liver lesions a combination of portal vein embolization and CD133^+^ BMSC administration increased the degree of hepatic regeneration in comparison with embolization alone [85]. In 9 patients with cirrhosis, who received portal vein infusion of unsorted autologous BMSCs, an improvement in Child-Pugh score and albumin levels was reported [86]. Recently, a significant increase of liver function postLR has been documented in patients with cirrhosis and hepatocellular carcinoma, following autologous BMSC transplantation prior to surgery [87]. So far, only one negative result regarding BMSC-therapies for end-stage liver disorders has been reported: in a phase-I clinical trial on decompensated cirrhotic patients, the infusion of autologous CD34^+^BM cells through the hepatic artery was unsafe and ineffective in improving the liver function [88].

### 5.2. Circulating Hematopoietic Stem Cell Mobilization, Collection and Reinfusion

Other SC-based therapies in hepatology have been based upon the collection from peripheral blood by leukapheresis and subsequent reinfusion of circulating HSCs, mobilized by G-CSF administration. The feasibility, safety, and pattern of BMSC mobilization with G-CSF in patients affected by cirrhosis has been evaluated [89]. Yannaki et al. described boost infusions of mobilized CD34^+^ cells after a standard G-CSF regimen in two patients. The procedure was safe, well tolerated and associated with a lasting amelioration in the clinical course of the disease during the follow-up (1 month) [90]. A significant biochemical and histopathological improvement was achieved by intraportal administration of mobilized CD34^+^ BMSCs following G-CSF exposure in one patient affected by drug-induced acute liver failure [91]. In a phase-I clinical trial on 5 patients with acute on chronic liver failure, G-CSF administration, followed by collection and intraportal or intrahepatic reinfusion of circulating CD34^+^ cells, resulted in an improvement of the hepatic function in more than 50% of the cases, without significant side effects during a follow-up of 60 days [92]. The same patients were then monitored for up to 18 months: the procedure was safe in short and over long term, by absence of tumor formation and the beneficial effects lasted around 12 months [93]. Recently, in 9 patients with alcohol-related cirrhosis, the reinfusion into the hepatic artery of CD34+ BM-derived cells, collected after G-CSF mobilization and *in vitro* expanded, was well-tolerated and produced a clinical and biochemical improvement [94]. In another trial, a total of 40 patients with HBV-related cirrhosis were randomized to receive G-CSF alone or in combination with leukapheresis and reinfusion of peripheral blood monocytes into the hepatic artery. During a follow-up of 6 months, a significant biochemical and clinical improvement was observed in both groups, even though the subjects receiving G-CSF *plus* SC infusion obtained the greater and longer-lasting beneficial effects [95].

### 5.3. G-CSF Treatment

Given its beneficial role in hepatic regeneration, G-CSF alone has been employed for the treatment of end-stage liver diseases in humans. Gaia et al. treated 8 patients affected by severe liver cirrhosis with G-CSF 5 *μ*g/Kg bid for three days: the treatment was well tolerated in all patients during a follow-up of 8 months, and a mobilization of BMSCs coexpressing epithelial and stem markers was noted [89]. Our group administered G-CSF (5 or 15 microgr/Kg/day) for 6 days to 24 patients with severe liver cirrhosis. This procedure was safe, resulted in a dose-dependent mobilization of BMSCs, but did not achieve any significant clinical improvement [96]. Similarly, Lorenzini et al. treated 18 nondecompensated cirrhotic patients with G-CSF, obtaining a good CD34^+^/CD133^+^ cell mobilization, despite the absence of clinical improvement [97]. Spahr et al. recently published the largest randomized trial, conducted on 24 patients with alcoholic cirrhosis, randomized to standard care associated with G-CSF or standard care alone. G-CSF was safe and able to mobilize CD34^+^ cells and increase HGF; however, the study was too small to make any comment regarding survival or efficacy. Interestingly, G-CSF was associated with the induction of HPC proliferation within 7 days of administration [98]. 

Most of the above-mentioned clinical trials share common limits, being conducted on small groups of patients, without controls, and using outcome parameters easily subjected to be biased, as reviewed elsewhere [5, 7, 23, 99–103]. Overall, the use of BMSCs for the treatment of end-stage liver diseases holds several advantages, such as easy accessibility, unlimited supply and no risks of rejection or need for immunosuppressive therapies, when autologous cells are employed. Nonetheless, some conceptual issues still limit the diffusion of such treatments in the clinical practice.

(1) On the basis of the previously reported preclinical data, BM cells seem to facilitate liver regeneration mainly by a microenvironment modulation, which is likely to be transitory. In such a case, multiple treatments would presumably be required to achieve significant and lasting clinical results; technical issues that need to be addressed regard the surface antigens used for HSC purification, the route of delivery, the amount of infused cells and the timing of infusions.
(2) It is not clear at present whether mobilization with G-CSF without leukapheresis and reinfusion of HSCs would suffice to gain a significant clinical benefit. Moreover, G-CSF therapies need to be standardized in terms of dosage, timing, and eventually association with other cytokines or interventions.
(3) The possibility of cell fusion and the risk of malignant transformation of the transplanted cells, especially if *in vitro* pre-expanded before reinfusion, cannot be excluded and impose a careful evaluation and longer follow-up periods for assessing the safety and efficacy of SC-based treatments.
(4) BMSCs have the potential to differentiate into endothelial cells and fibroblasts within the liver, and, as such, they might exert a profibrogenic effect [57]. Therefore, it is mandatory to examine the involvement of the infused SCs in the reconstitution of hepatic nonparenchymal cells.

Until these open questions can be properly answered, through an intense collaborative effort from basic cell biologists, translational scientists and clinicians, SC-based therapies for liver diseases should be limited to well designed and adequately powered clinical trials. 

## 6. Conclusions

SCs are promising tools at the service of regenerative medicine for the treatment of degenerative disorders, inborn errors of metabolism, and organ failure. In hepatology, the first attempts to translate SC basic research into new clinical strategies for the treatment of acute and chronic hepatopathies have been made. In particular, HSCs transplantation and G-CSF infusion are an attractive option for the treatment of end stage liver pathologies, as we already handle G-CSF and transplant HSCs in clinics, for hematologic and oncologic disorders. Right now, the major role for stem cell therapy is as a bridge to transplantation or as a way of maintaining those patients who are not eligible for OLT. Nonetheless, critical aspects need to be further addressed, including the long-term safety, tolerability, and efficacy of these SC-based treatments, as well as their carcinogenic potential. As a consequence, it is paramount to conduct larger and well-designed clinical trials to fully establish the safety profile of such therapies and to define the target patient groups with efficacy assessed by standardised protocols.
